# Epidermal growth factor receptor is associated with the onset of skeletal related events in non-small cell lung cancer

**DOI:** 10.18632/oncotarget.18759

**Published:** 2017-06-28

**Authors:** Shu-Mei Huang, Jin-Ji Yang, Hua-Jun Chen, Si-Pei Wu, Xiao-Yan Bai, Qing Zhou, Hai-Yan Tu, Yi-Long Wu

**Affiliations:** ^1^ Southern Medical University, Guangzhou, Guangdong, P.R. China; ^2^ Guangdong Lung Cancer Institute, Guangdong General Hospital & Guangdong Academy of Medical Sciences, Guangzhou, Guangdong, P.R. China

**Keywords:** non-small cell lung cancer, epidermal growth factor receptor, tyrosine kinase inhibitors, bone metastasis, skeletal related events

## Abstract

**Background:**

Bone metastasis and skeletal related events (SREs) are common in non-small cell lung cancer (NSCLC). Patients with mutant epidermal growth factor receptor (EGFR) could benefit from tyrosine kinase inhibitors (TKIs). However, it is unclear whether SRE is influenced by EGFR status. We aimed to evaluate the correlation of EGFR status and TKIs with the incidence of SREs.

**Methods:**

We conducted a retrospective study of stage IV NSCLC patients with bone metastasis. Incidence rate of SREs was collected and was compared using chi-square test. Logistic-regression analysis was used to identify the risk factors predicting the incidence of SREs.

**Results:**

410 eligible patients were enrolled in the study. 49.0% were detected with EGFR mutation. 49.8% of patients received EGFR-TKIs therapy prior to the onset of SREs. 42.7% experienced at least one SRE. Patients who were treated with TKIs held lower incidence of SREs than patients who were not treated with TKIs (23.5% vs 61.7%, *p<0.001*). Multivariate analysis showed that poor performance status (OR 5.550, 95%CI 2.290-13.450; *p<0.001*) and mutant EGFR (OR 3.050, 95%CI 1.608-5.787, *p=0.001*) were independent risk factors predicting the onset of SREs, while the usage of TKIs (OR 0.102, 95%CI 0.054-0.193, *p<0.001*) was a protective factor of SREs in NSCLC patients with bone metastasis.

**Conclusions:**

This study indicates that the incidence of SREs is common in both patients with and without EGFR mutation. Poor performance ability and mutant EGFR imply higher risks of SREs, while the usage of TKIs may be a protective factor of SREs.

## INTRODUCTION

Bone metastasis was observed in 30% to 60% of advanced non-small cell lung cancer (NSCLC) patients [1, 2], subsequently 30% to 65% would experience at least one skeletal related event (SRE) during follow-up [2–4]. SREs were defined as pain requiring palliative radiation, bone instability requiring palliative surgery, pathological fracture, and spinal compression [1]. In clinical practice, the early diagnosis of bone metastasis and the prevention of SREs in NSCLC patients were usually overlooked, owing to a relatively short life expectancy. For patients with unknown driver gene mutation status, survival post bone metastasis was reported to be less than 6 months [2, 5]. For patients with mutant epidermal growth factor receptor (EGFR), it was a different story. Median survival was about 3.5 years for patients with mutant EGFR and who received targeted therapy, while median survival was about 2.4 years for patients with mutant driver gene who did not received tyrosine kinase inhibitors (TKIs) [6–8]. For patients with mutant EGFR who received TKIs, concerns aroused whether the activity of the EGFR signal pathway in bone microenvironment would expedite the onset of SREs, or could TKIs therapy inhibit bone invasion and decrease the incidence of SREs. Limited studies are available to illustrate factors predicting SREs in NSCLC patients with known EGFR status [9, 10], precluding a definite conclusion. This retrospective study was to explore the correlation of EGFR status and the usage of TKIs with the incidence of SREs in NSCLC patients with bone metastasis.

## RESULTS

### Baseline characteristics

Among patients who were diagnosed with stage IV NSCLC from 2003 to 2012 in our institute, 63.9% of patients with mutant EGFR were detected to have bone metastasis, while 54.4% of patients with wild type EGFR were found to have bone involvement (*p = 0.004*). 410 eligible patients were enrolled in our study, of whom 40.2% were female, and 60.2% were never smokers. At the time of bone metastasis, 90.5% of patients presented ECOG PS of 0 or 1. Compared to patients with wild type EGFR, patients with mutant EGFR presented higher percentage of PS 0 or 1 (95.0% *vs* 86.1%, *p = 0.002*). Adenocarcinoma was the most common type of histology, consisting of 88.8%, and EGFR mutation was detected in 49.0% of patients. Systemic therapy was administered to 99% of patients who were included in our study. 49.8% of patients received EGFR-TKIs prior to the onset of first SRE. For patients harboring EGFR mutation, 63.7% received TKIs as first line treatment. Table 1 showed patient characteristics in this study.

**Table 1:** Clinical characteristics of NSCLC patients with bone metastasis which were grouped by EGFR mutation status

	EGFR mutant (%) *N*=201	EGFR wild type (%) *N*=209	*p* value
Gender Female male	106(52.7) 95(47.3)	59(28.2) 150(71.8)	*<0.001*
Age Median (range)(year)	59.0 (28-85)	58.0 (24-86)	*0.325*
ECOG PS 0, 1 2, 3	191(95.0) 10(5.0)	180(86.1) 29(13.9)	*0.002*
Smoking history Nonsmoker Smoker	152(75.6) 49(24.4)	95(45.5) 114(54.5)	*<0.001*
Pathology ADC Others	196(97.5) 5(2.5)	168(80.4) 41(19.6)	*<0.001*
TKIs therapy No Yes	41(20.4) 160(79.6)	165(78.9) 44(21.1)	*<0.001*
Number Solitary Multiple	32(15.9) 169(84.1)	46(22.0) 163(78.0)	*0.116*
Density OB OC Mixed type NA	61(30.3) 119(59.2) 21(10.4) -	51(24.4) 142(67.9) 15(7.2) 1(0.5)	*0.149*
Bisphosphonates Yes No	57(28.4) 144(71.6)	62(29.7) 147(70.3)	*0.771*
SREs No Yes	126(62.7) 75(37.3)	109(52.2) 100(47.8)	*0.031*

Clinical characteristics of NSCLC patients with bone metastasis which were grouped by EGFR mutation status. EGFR, epidermal growth factor receptor; mutant EGFR was confined to exon 19 deletion and exon 21 L858R point mutation in our study; ECOG PS, Eastern Cooperative Oncology Group Performance Status; ADC, adenocarcinoma; TKIs, tyrosine kinase inhibitors, usage of TKIs was defined as usage prior to first SRE; OB, osteoblastic lesion; OC, osteolytic lesion; NA, data not available; SREs, skeletal related events; Quantitative variables were compared using student T test and qualitative variables were compared using chi-square test. *P* value lowered than 0.05 at two tails was considered to be statistically significant.

### Characteristics of bone lesions and SREs

81.0% of patients presented bone involvement at the time of the diagnosis of NSCLC. For patients without bone involvement at initial diagnosis of advanced NSCLC, the median time to bone metastasis was 11 months (95% confidence interval (CI) 7.4m-14.6m). At the time of bone metastasis, 81% had multiple bone lesions and 63.7% had osteolytic lesions, which were not significantly different between patients with and without EGFR mutation (*p = 0.116*, *p = 0.149*; respectively). Bisphosphonates were given to patients according to patients’ clinical manifestations. Bisphosphonates were administered in 29% of patients (zoledronic acid consisted of 28.5%) prior to SREs, which was similar between patients with and without EGFR mutation (*p = 0.771*). 52 out of 118 patients were given at the time of bone involvement, and 66 out of 118 patients were given during follow up, when patients complained about symptoms. Patients who received bisphosphonates prophylactically confronted a lower rate of SRE comparing with patients who did not (36.1% *vs* 45.4%, *p = 0.087*). Besides, in our study, bisphosphonates were used in 41 patients after the onset of the first SRE.

Median time of follow up was 16 months. 42.7% of patients confronted at least one SRE. For patients with EGFR mutation, the incidence rate was 37.3%, which was about 10% lower than patients with wild type EGFR (*p = 0.031*). For patients who were treated with TKIs, regardless of EGFR status, 23.5% experienced at least one SRE, while for patients without TKIs therapy, 61.7% experienced at least one SRE (*p < 0.001*). We should notice that 13.7% of patients were initially diagnosed with NSCLC because of the occurrence of SREs, and 4.9% were not diagnosed with bone involvement until the onset of SRE during follow up.

Palliative radiotherapy was the most common type of SRE, consisting of 50.3%, and pathological fracture was the second most common type, about 30.0%. The difference of manifestations of SREs was not significant between patients with and without EGFR mutation (*p = 0.421*). Table 2 showed types of SREs for patients who were included in our study.

**Table 2:** Types of first SRE in NSCLC patients

	EGFR mutant (%) *N*=75	EGFR wild type (%) *N*=100	*p* value
Radiotherapy	39(52.0)	49(49.0)	*0.421*
Surgery	3(4.0)	7(7.0)	
Fracture	26(34.7)	28(28.0)	
Compression	7(9.3)	16(16.0)	

Types of first SRE in NSCLC patients. Radiotherapy, pain requiring palliative radiation; Surgery, bone instability requiring palliative surgery; Fracture, pathological fracture; Compression, spinal compression. *P* value was obtained using chi-square test.

### Factors predicting SREs for NSCLC patients with bone metastasis

Table 3 showed the variables that were correlated to the onset of first SRE in NSCLC patients with bone metastasis. In univariate analysis, ECOG PS, EGFR mutation status, treatment strategies, density of bone lesions and usage of bisphosphonates were conducted with a p value lowered than 0.10, thus were included in multivariate analysis. The results of multivariate analysis showed that poor PS (OR 5.550, 95%CI 2.290-13.450; *p < 0.001*) and mutant EGFR (OR 3.050, 95%CI 1.608-5.787, *p = 0.001*) were independent risk factors predicting the onset of SREs, while the usage of TKIs (OR 0.102, 95%CI 0.054-0.193, *p < 0.001*) was a protective factor.

**Table 3:** Univariate and multivariate analysis of factors predicting SREs for NSCLC patients

	univariate OR	95%CI	analysis *p* value	multivariate OR	95%CI	analysis *p* value
Gender Female/male	1.363	0.912-2.039	*0.131*	-	-	-
Age <60y/≥60y	0.899	0.608-1.331	*0.595*	-	-	-
ECOG PS 0,1/2,3	6.109	2.732-13.660	*<0.001*	5.550	2.290-13.450	*<0.001*
Smoking history Nonsmoker/smoker	1.202	0.806-1.792	*0.367*	-	-	-
Pathology ADC/others	0.938	0.503-1.749	*0.841*	-	-	-
EGFR status Wild type/mutant	0.649	0.437-0.962	*0.031*	3.050	1.608-5.787	*0.001*
TKIs therapy No/yes	0.191	0.125-0.294	*<0.001*	0.102	0.054-0.193	*<0.001*
Number Solitary/multiple	1.519	0.908-2.541	*0.111*	-	-	-
Quality OB/OC OB/mixed type	2.208 1.297	1.380-3.533 0.588-2.858	*0.003* *0.001* *0.519*	1.629 1.097	0.970-2.737 0.451-2.668	*0.150* *0.065* *0.838*
Bisphosphonate Yes/no	1.467	0.945-2.277	*0.087*	0.923	0.553-1.541	*0.761*

410 patients were included in the analysis. Factors with a *p* value lower than 0.10 in univariate analysis were included in multivariate analysis. In multivariate analysis, factors with a *p* value lower than 0.05 were considered independent risk factors predicting SRE. Poor PS and mutant EGFR were considered as independent risk factors predicting SREs in NSCLC patients, while the usage of EGFR-TKIs was considered as a protective factor of SRE in NSCLC patients with bone metastasis. Logistic regression model was conducted to analyzed potential factors predicting SREs.

Subgroup analysis of 354 patients who did not experience initial SRE at the time of NSCLC diagnosis showed that the difference of SRE incidence was not significant between patients with and without EGFR mutation (29.6% *vs* 37.7%, *p = 0.107*). In subgroup analysis, patients who were treated with TKIs presented lower incidence of SREs than patients who were not treated with TKIs did (23.5% *vs* 47.3%, *p < 0.001*). For patients who did not experience initial SRE, the usage of TKIs was a protective factor of SREs (OR 0.342, 95%CI 0.217-0.540, *p < 0.001*).

## DISCUSSION

For NSCLC patients who harbored sensitive EGFR mutation, TKIs tremendously improved their quality of life and prolonged their life expectancy. Considering that the EGFR signal pathway was not only associated with angiogenesis [14], activating cell proliferation and epidermal mesenchymal transition in lung cancer cells [15], but also was related to bone matrix cell proliferation and differentiation [16], and that bone was a common site of distant involvement in NSCLC, we investigated bone metastasis and SREs among patients with known EGFR status. Confavreux and colleagues had reported that the incidence rate of bone metastasis was higher in EGFR-positive patients, comparing to the average rate in all stage IV NSCLC patients [17]. Our study showed that bone metastasis was more common in EGFR mutant patients than in EGFR wild type patients. We found that the distribution of bone lesions and types of SREs were similar between patients with and without EGFR mutation. With respect to performance status, patients with mutant EGFR possessed better performance status at the time of bone presentation than patients with wild type EGFR did. The incidence rate of SREs was lower in patients who were treated with EGFR-TKIs. Poor performance status and positive EGFR mutation were independent negative factors predicting SREs. The usage of EGFR-TKIs could decrease the onset of SREs in NSCLC patients.

In our study, 49% of patients were detected with mutant EGFR, who possessed better performance ability at the time of bone metastasis. Subgroup analysis showed that was significant in patients who had bone lesions at the time of diagnosis of NSCLC (*p = 0.005*), but was not significant in patients who developed bone lesions during follow up (*p = 0.165*) (data not shown). We assumed that, for one hand, TKIs impeded the aggressive behavior of tumor cells and thus improved the quality of life [18]. While for patients who developed bone lesions during follow up, the appearance of bone lesions indicated that tumor cells developed resistance to targeted drugs and progressed aggressively.

Recently, the characteristics of isolated bone failure (IBF) without progression in extraskeletal organs in EGFR mutation patients were evaluated by Hwang and colleagues [19]. They found that IBF were more frequently detected in patients who responded to EGFR-TKIs compared to those without clinical benefit (54.4% *vs*14.3%, *p = 0.007*), in patients with good performance ability (82.5% *vs* 42.9%, *p = 0.005*), and in patients with exon 19 deletion (68.4% *vs* 35.7%, *p = 0.024*). And clinical benefit from EGFR-TKIs was the independent risk factor predicting IBF (OR 6.647, 95%CI 1.328-33.262, *p = 0.021*). Their results indicated that patients with IBF tended to have longer survival. The results revealed from their data and this study implied that EGFR-TKIs might affect the bone lesions separately from lesions in other organs, which awaited more findings to identify the possibility.

We then evaluated the difference of SREs in patients with known EGFR status. It revealed that mutant EGFR independently indicated higher risk of SREs, while the administration of EGFR-TKIs, regardless in mutant or wild type patients, was a protective factor of SREs. Several studies have previously analyzed the correlation. Aiba and colleagues analyzed 47 patients who were diagnosed with NSCLC with bone lesions in spinal column, among which 41 patients were detected with known EGFR status. Patients with EGFR mutation intended to have a lower risk to experience SREs comparing to patients without EGFR mutation (OR 0.61, 95%CI 0.38-0.99) [12]. Sun and colleagues reported that smokers, nonadenocarcinoma, poor performance status (ECOG PS≥2), and no history of EGFR TKIs therapy were independent risk factors of development of SREs throughout the course of disease [9]. In a prospective study, however, risk of SREs was not significantly associated with the history of EGFR-TKIs therapy [13]. Summary of studies of relatively large studies that evaluated the correlation of EGFR and TKIs with SREs was shown in Table 4. We should notify that in these studies, the sample size was relatively small, and that the EGFR mutation status was not detected in most patients who were treated with EGFR-TKIs. The results cannot be widely applied. The result of this study, however, indicated that mutant EGFR in lung cancer cells prompted the destruction of bone lesions, leading to higher risk of SREs. However, a history of TKIs therapy disrupted the interaction, generating lower incidence of SREs. Several aspects support our hypothesis. As we has discussed before that EGFR mutant patients possessed better performance status would be a partial explanation. Also, the interaction of EGFR signaling pathway and TKIs in both tumor cells and bone matrix cells was salient.

**Table 4:** Summary of the studies of relatively large studies that evaluated the correlation of EGFR and TKIs with SREs in NSCLC patients

Author	Type	*N*	Incidence of SRE	Risk of SREs (EGFR)	Risk of SREs (EGFR-TKIs)
Huang	Retrospective China	410 201(EGFR+) 209 (EGFR wt)	37.3% 47.8%	OR 3.050 (95%CI 1.608-5.787) *p*=0.001	OR 0.102 (95%CI 0.054-0.193) *p*<0.001
Hendriks [11]	Retrospective Netherlands	102 37(EGFR+) 34(KRAS+) 31(WT)	51.4% 64.7% 48.4%		
Aiba [12]	Retrospective Japan	41 17(EGFR +) 24 (wt)	17.6% 45.8%	OR 0.61 (95%CI 0.38-0.99) *p*=0.060	OR 0.88 (95%CI 0.48-1.60) *p*=0.680
Sun [9]	Retrospective Korea	273	62.6%		OR 0.64 (95%CI 0.35-1.18) *p*=0.16
Katakami [13]	Prospective Japan	59	33.9%		HR 0.75 (95%CI 0.27-2.04) *p*=0.569

Summary of the studies of relatively large studies that evaluated the correlation of EGFR and TKIs with SREs in NSCLC. EGFR +, EGFR mutation; wt, EGFR wild type; KARS +, KRAS mutation; WT, both EGFR and KRAS wild type; N, NSCLC patients with bone metastasis were included; CI, confidence interval.

In the bone microenvironment, active EGFR signal pathway in tumor cells was associated with angiogenesis and cell proliferation. Tumor cells could impair bone matrix directly. In addition, the EGFR signal pathway could disrupt the balance of bone matrix cells indirectly. Studies showed that the EGFR network was vital in bone biology [16]. EGFR signal pathway promoted the mesenchymal cells to differentiate into osteoclasts, activated the osteoclasts, thus accelerated bone absorption. These could partially explain why mutant EGFR in lung cancer indicated higher risk of SREs.

On the other hand, EGFR-TKIs were reported to inhibit both tumor cells and the crosstalk between tumor cells and bone matrix cells [20]. It was demonstrated by several studies that TKIs disrupted the interaction between tumor cells and mesenchymal stem cells and osteoclastic cells. Furugaki and colleagues validated that erlotinib inhibited osteolytic bone invasion which was induced by human NSCLC cell line NCI-H292 (EGFR wild type cell line) in SCID mice [21]. They found that the expression level of receptor activator of NF-κB ligand (RANKL) was decreased to the basal level of the non-tumor bearing mouse, and the activation of osteoclastic cells was decreased after adding erlotinib. Unfortunately, the influence of EGFR mutant NSCLC tumor cells imposed on the bone niches was not clear in this study, as the HCC827 NSCLC cell line (EGFR mutant cell line) bone metastatic model was not established. Garfield indicated that TKIs could decrease the secretion of growth factor and RANKL, and thus induced the differentiation of osteoblast and inhibited the proliferation and activity of osteoclast [22, 23]. Similar results were shown in SCLC cell lines [24] and prostate cancer cell lines [25].

Interestingly, clinical practice had witnessed the result of crosstalk interruption. Osteoblastic flare was reported in patients who were treated with EGFR TKIs [26–28], which represented as a healing response of the osteolytic lesions or appearance of osteoblastic lesions while lesions in other sites were well controlled. These data revealed that TKIs could inhibit the activity of osteoclasts and activate the osteoblast, thus could interrupt the vicious cycle of tumor cells and bone matrix cells in the microenvironment, and decreased the risk of SREs. In our institute, as the relevant data was not required to collect during follow up, the proportion of healing response was not available in our study.

To integrate, we found that SREs were common in both patients with and without EGFR mutation. Positive prophylactic process is recommended to impose on patients with high risks to experience SREs, and to reduce the occurrence of SREs. To EGFR mutant patients, EGFR-TKI is recommended as first line therapy. Combined with our results, it implies that patients with bone metastasis who would receive TKIs would hold lower risks of SREs compared to patients who would not.

Our study has limitations. First of all, it is a retrospective study in nature. Secondary, as patients included in this study were extracted from a single institute, the probably existing Berkson bias limits the application of the results to the nationwide population. The diagnostic methods of bone involvement were not standardized. However, each method exposes its limitation. In clinical practice, bone screening was not required until patients presenting with bone pain or SREs during follow up [1]. In our study, EGFR mutation was constrained to EGFR exon 19 deletion and exon 21 L858R point mutation, while patients with EGFR uncommon mutation and driver gene mutations other than EGFR were not considered in the study. Further studies focusing on these driver gene mutations are needed.

## CONCLUSIONS

This study indicates that SREs are common in NSCLC patients with and without EGFR mutation. Poor performance ability and mutant EGFR imply higher risks of SREs, while the usage of TKIs may be a protective factor of SREs.

## MATERIALS AND METHODS

### Patients

We retrospectively reviewed clinic-pathological data of consecutive patients who were diagnosed with stage IV NSCLC with bone metastasis in Guangdong General Hospital from 2003 January 1^st^ to 2012 December 31^st^.

Patients would be included in this study if they fulfilled the following criteria, histology of NSCLC was confirmed pathologically or cytologically based on 2004 World Health Organization (WHO) classification of lung cancer [29], and the new adenocarcinoma classification [30]; known EGFR mutation status was detected by a polymerase chain reaction (PCR) based direct sequencing (SANGER sequencing) method [31] or amplification refractory mutation system (ARMS) [32] (sensitive mutation was defined as exon 19 deletion or exon 21 L858R point mutation); bone involvement was confirmed by at least one of the following methods, magnetic resonance image (MRI), bone marrow scintigraphy (ECT), positron emission tomography (PET), computed tomography (CT), X-ray, or biopsy [33, 34].

Patients would be excluded if they encountered one or more of the followings, diagnosis of secondary primary carcinoma within 2 years of the diagnosis of NSCLC; mutation of driver genes other than EGFR was detected, such as ALK, c-MET, ROS-1, KRAS.

Data of evaluated variables, including gender, age, smoking history, Eastern Cooperative Oncology Group Performance Status (ECOG PS), histology, EGFR mutation status, number and density of bone metastasis, usage of bisphosphonates, time to bone metastasis, SREs types, and treatment strategy prior to SREs, were collected and analyzed.

### Ethic statement

The study was approved by the institutional review boards of Guangdong General Hospital. Furthermore, a written consent was deemed unnecessary because the data was retrospectively reviewed, and the treatment strategy was not influenced by this study.

### Statistics

Descriptive statistics were conducted to analyze demographic variables and incidence of SREs. Clinical-pathological parameters were estimated as possible factors to predict incidence of SREs using a logistic regression model. Patients without a documented date of events were censored at the date of death or date of last follow-up. Parameters with a p value lowered than 0.10 in univariate analysis were included in multivariate analysis. P value lowered than 0.05 at two tails was considered of statistical significance. All statistics were analyzed by SPSS (IMB statistics, version 20.0).

## Abbreviations

NSCLC: non-small cell lung cancer
EGFR: epidermal growth factor receptor
TKIs: tyrosine kinase inhibitors
SREs: skeletal related events
ECOG PS: Eastern Cooperative Oncology Group Performance Status
OB: osteoblastic
OC: osteolytic
ALK: anaplastic lymphoma kinase
RANKL: receptor activator of nuclear factor kappa-B ligand
OR: odd ratio
HR: hazard ratio
CI: confidence interval
*vs*: *versus*
